# Characterizing interactions of *Leptospira interrogans* with proximal renal tubule epithelial cells

**DOI:** 10.1186/s12866-018-1206-8

**Published:** 2018-07-04

**Authors:** Takayoshi Yamaguchi, Naomi Higa, Nobuhiko Okura, Arina Matsumoto, Idam Hermawan, Tetsu Yamashiro, Toshihiko Suzuki, Claudia Toma

**Affiliations:** 1 Department of Bacteriology, Graduate School of Medicine, University of the Ryukyus, Okinawa, 903-0215 Japan; 2 Department of Molecular Anatomy, Graduate School of Medicine, University of the Ryukyus, Okinawa, 903-0215 Japan; 3 Department of Bacterial Pathogenesis, Infection and Host Response, Graduate School of Medicine and Dental Sciences, Tokyo Medical and Dental University, Tokyo, 113-8510 Japan; 4 Present address: Department of Food and Nutrition Science, Junior College, Sagami Women’s University, Sagamihara, Kanagawa 252-0383 Japan; 5 Present address: Okinawa Industrial Technology Center, Okinawa, 904-2234 Japan

**Keywords:** *Leptospira interrogans*, Kidney, Colonization, Proximal tubule, Renal epithelial cell

## Abstract

**Background:**

*Leptospira interrogans* is a pathogenic, spirochetal bacterium that is responsible for leptospirosis, an emerging worldwide zoonosis. Leptospires colonize the renal proximal tubules and chronically infect the kidney. Live bacteria are excreted into urine, contaminating the environment. While it is well known that leptospires can persist in the kidneys without signs of disease for several months, the interactions of leptospires with the proximal renal epithelial tubule cells that allow the chronic renal colonization have not been elucidated yet. In the present study, we compared the interactions between a virulent, low passage (LP) strain and a cultured-attenuated, high passage (HP) strain with renal proximal tubule epithelial cells (RPTECs) to elucidate the strategies used by *Leptospira* to colonize the kidney.

**Results:**

Kinetics analysis of kidney colonization in a mouse model of chronic infection performed by quantitative real-time PCR and immunofluorescence, showed that the LP strain reached the kidney by 3 days post infection (pi) and attached to the basal membrane side of the renal epithelial cells. At 10 days pi, some leptospires were attached to the luminal side of the tubular epithelia and the number of colonizing leptospires gradually increased. On the other hand, the HP strain was cleared during hematogenous dissemination and did not colonize the kidney. Transmission electron microscopy analysis of LP-infected kidneys at 25 days pi showed aggregated leptospires and membrane vesicles attached to the epithelial brush border. Leptospiral kidney colonization altered the organization of the RPTEC brush border. An in vitro model of infection using TCMK-1 cells, showed that leptospiral infection induced a host stress response, which is delayed in LP-infected cells.

**Conclusions:**

After hematogenous dissemination, leptospires create protective and replicative niches in the base membrane and luminal sides of the RPTECs. During the long-term colonization, leptospires attached to the RPTEC brush borders and membrane vesicles might be involved in the formation of a biofilm-like structure in vivo. Our results also suggested that the virulent strain is able to manipulate host cell stress responses to promote renal colonization.

## Background

Leptospirosis is a widespread zoonotic disease caused by *Leptospira interrogans* and other pathogenic *Leptospira* species. The disease is epidemic in Asia, South America and Oceania but considered as an emerging infectious disease in Europe, North America and Africa [1, 2]. Leptospires enter the host through the skin or mucous membranes, disseminate hematogenously, and then reach target organs, such as the liver, lungs and, mainly, the renal proximal tubules (RPTs) in the kidneys, where they can survive for several months [3]. Leptospiremia in the patients as well as the animal hosts after infection occurs once, at the early stage of infection, for approximately 3 to 7 days [4].

In vitro studies have shown that pathogenic leptospires can evade host defense mechanisms by surviving within macrophages, delaying phagosome maturation [5], and can resist to reactive oxygen species [6]. Pathogenic leptospires can also recruit soluble complement regulators, thus preventing activation and formation of a lytic membrane attack on its surface, leading to its successful dissemination to the target organs [7]. Recently, in vivo imaging systems were used to analyze the distribution of leptospires in the whole bodies of infected animals during the early stages of infection as well as in late colonization. These studies revealed that at the early stage of infection, adipose tissue is the colonization site, where leptospires grow before hematogenous dissemination [8]. The majority of leptospires are cleared during dissemination, but a small number of bacteria that successfully evade host defense mechanisms, colonized the kidneys where they are able to replicate and persist despite being constantly shedding into the urine [9]. A wide range of mechanisms possibly involved in the ability of leptospires to survive in the kidneys, such as biofilm formation, have been suggested [10]. Live imaging of bioluminescent bacteria and quantitative real-time PCR have been used to characterize the kinetics of kidney colonization in various animal infection models [9, 11, 12], however, the kinetics of proximal renal tubule colonization and the mechanisms that allow the long-term persistence of bacteria in the tubules remains poorly understood.

In the hamster model of infection, leptospires in the interstitial space have been reported to induce necrosis of the epithelial cells, while no epithelial cell death was observed in a mouse model of infection [13]. These differences in epithelial cells outcome might be a result of the differential host inflammatory response during chronic infection in both animal models [13]. However, pathogen-driven strategies to promote persistence in renal proximal tubule epithelial cells (RPTECs) could not be excluded.

Ratet et al. [9] have reported that the renal colonization protects leptospires from blood defenses and antibiotic treatment. In leptospirosis patients, timely treatment with antibiotics is effective and may dramatically rescue patients from multiple organ failure. On the other hand, chronic leptospirosis may also develop in humans if bacteria persist in the tubular lumen after acute leptospirosis [14]. Chronic asymptomatic leptospirosis has recently been reported in a Peruvian population and in Taiwan, suggesting that the renal colonization is not a peculiarity of some animal carriers [14, 15]. Since antibiotic treatment would not be effective in these asymptomatic carriers, a better understanding of the interaction of leptospires with RPTECs is needed to develop novel strategies for controlling the disease. Hence, in this work we used in vivo and in vitro models of infection to elucidate the strategies used by a virulent *L. interrogans* serovar Manilae strain to interact with RPTECs and persist in the kidneys.

## Methods

### Bacterial strains and culture

Leptospires were cultured in Ellinghausen–McCullough–Johnson–Harris (EMJH) broth (Difco) at 30 °C. *L. interrogans* serovar Manilae strain UP-MMC-NIID isolated from the blood of a human patient with severe leptospirosis [16] was propagated in specific pathogen-free C3H/HeJ mice to maintain pathogenicity and used as virulent, low-passage (LP) strain. The attenuated-high passage strain (HP) strain was generated by serially culturing the LP strain in liquid EMJH more than 60 times as described previously [17]. The attenuation of HP was confirmed in the CH3/HeJ mice infection model which is an in vivo murine model of severe leptospirosis [18].

### Animal infection

*L. interrogans* LP or HP strains in the exponential phase of growth were adjusted to 10^7^ cells in 0.5 mL of PBS (a sublethal dose) and injected intraperitoneally to C57BL/6 mice (female, 5-weeks old) purchased from Japan SLC (Tokyo, Japan). Mice were acclimated for at least 1 week in the animal facility with free access to food and water. The animals (*n* = 5 per group) were sacrificed at 1, 3, 10, 21 or 25 days pi by cervical dislocation and kidneys were quickly removed. One kidney was fixed in 4% formaldehyde for immunostaining and the other one was divided for use as material for DNA extraction and culture in EMJH broth to confirm the viability of colonizing leptospires. The kidney tissues were then embedded in tissue-embedding medium to ensure Optimal Cutting Temperature (O.C.T. compound, Sakura Finetek), frozen and sectioned with a Leica cryostat (model CM 1900).

### Quantitative real-time PCR (qPCR)

DNeasy Blood & Tissue Kit (Qiagen) was used for DNA extraction from the infected kidneys after mechanical disruption. Two microliters of DNA were added to a mixture containing Brilliant III SYBR Green QPCR master mix (Stratagene, Agilent Technologies) and 0.5 μM of both forward and reverse 16S rRNA gene primers as described previously [5]. The number of total bacteria was determined using a standard curve generated by the serial dilution of genomic DNA extracted from in vitro-cultivated bacteria and was expressed as the number of leptospires per 25 ng of total DNA extracted from kidneys.

### Cell culture and infection

TCMK-1 (ATCC^®^ CCL-139™) cells were grown in Dulbecco’s Modified Eagle Medium (DMEM) supplemented with 10% fetal bovine serum (FBS). The cells were seeded at 5 × 10^5^ cells per well in a six-well plate and maintained in a humidified incubator at 37 °C with 5% CO_2_ for 24 h before infection. The cells were infected with leptospires at a multiplicity of infection (MOI) of 100 per cell in prewarmed FBS-free DMEM. In the experiments using inhibitors, the TCMK-1 cells were incubated with 20 μM PARP inhibitor VIII, PJ34 (Calbiochem), 20 μM Z-VAD-FMK (R&D System) or 200 μM Trolox (Sigma). The plates were centrifuged at 500 g for 10 min to synchronize the stage of infection. TCMK-1 were then incubated at 37 °C in 5% CO_2_ and fixed at the appropriate time pi for immunostaining or TUNEL staining with 2% paraformaldehyde overnight at 4 °C. For crystal violet staining, cells were fixed with 4% paraformaldehyde at room temperature. For detection of reactive oxygen species, CellROX Green Reagent (Molecular Probes) was added to infected cells and incubated for 30 min for image acquisition using the Leica AF6500 widefield microscope.

### Immunofluorescence microscopy

Rhodamin-phalloidin (Molecular Probes) was used to visualized the actin filaments and TO-PRO-3 (Molecular Probes) was used to visualized the DNA. All secondary antibodies used in this study were from Jackson ImmunoResearch. Leptospires were stained with a rabbit polyclonal anti-*L. interrogans* antiserum (kindly provided by SYAM Villanueva, University of the Philippines) and labeled with anti-rabbit FITC, TRITC or Alexa647. For cubilin immunostaining, acetone-fixed sections were stained after epitope retrieval by heating in citrate buffer (10 mM sodium citrate [pH 6.0]), with anti-cubilin (R&D Systems, AF3700, sheep polyclonal) followed by the tyramide amplification method (TSA-Plus Fluorescein System; Perkin Elmer Life Sciences). Anti-AIF (Cell Signaling, D39D2 XP Rabbit mAb) was labeled with FITC-labeled secondary antibody. Stained samples were observed using a confocal laser scanning microscope (Leica TCS-SPE).

### Transmission electron microscopy (TEM)

For TEM analysis kidneys were pre-fixed in 2.5% glutaraldehyde in 0.1 M sodium cacodylate buffer (pH 7.4), and then were post-fixed in 1% OsO4 in the same buffer. The tissues were dehydrated in a graded ethanol series and embedded in epoxy resin (TAAB812). Thin sections were cut on an ultra-microtome with a diamond knife (MT-2C, RMC). The sections were double stained with uranyl acetate and lead citrate and examined with an electron microscope at 80 kV (H-750, Hitachi), at the University of the Ryukyus, Research Laboratory Center.

### Cell death assays

For the crystal violet assay, fixed cells were stained with 0.1% crystal violet solution. After extensive washing, the dye was eluted and analyzed at 595 nm. The TUNEL assay was performed with the Dead-End fluorometric TUNEL system (Promega) as recommended by the manufacturer.

### Statistical analyses

Statistical analyses were performed by the unpaired two-tailed Student’s *t* tests. Differences were considered significant at *P* < 0.05.

## Results

### Kinetics of leptospiral dissemination in mouse infected with low-passage number (LP) virulent or high-passage-number (HP) attenuated *Leptospira interrogans* strains

**Fig. 1:**
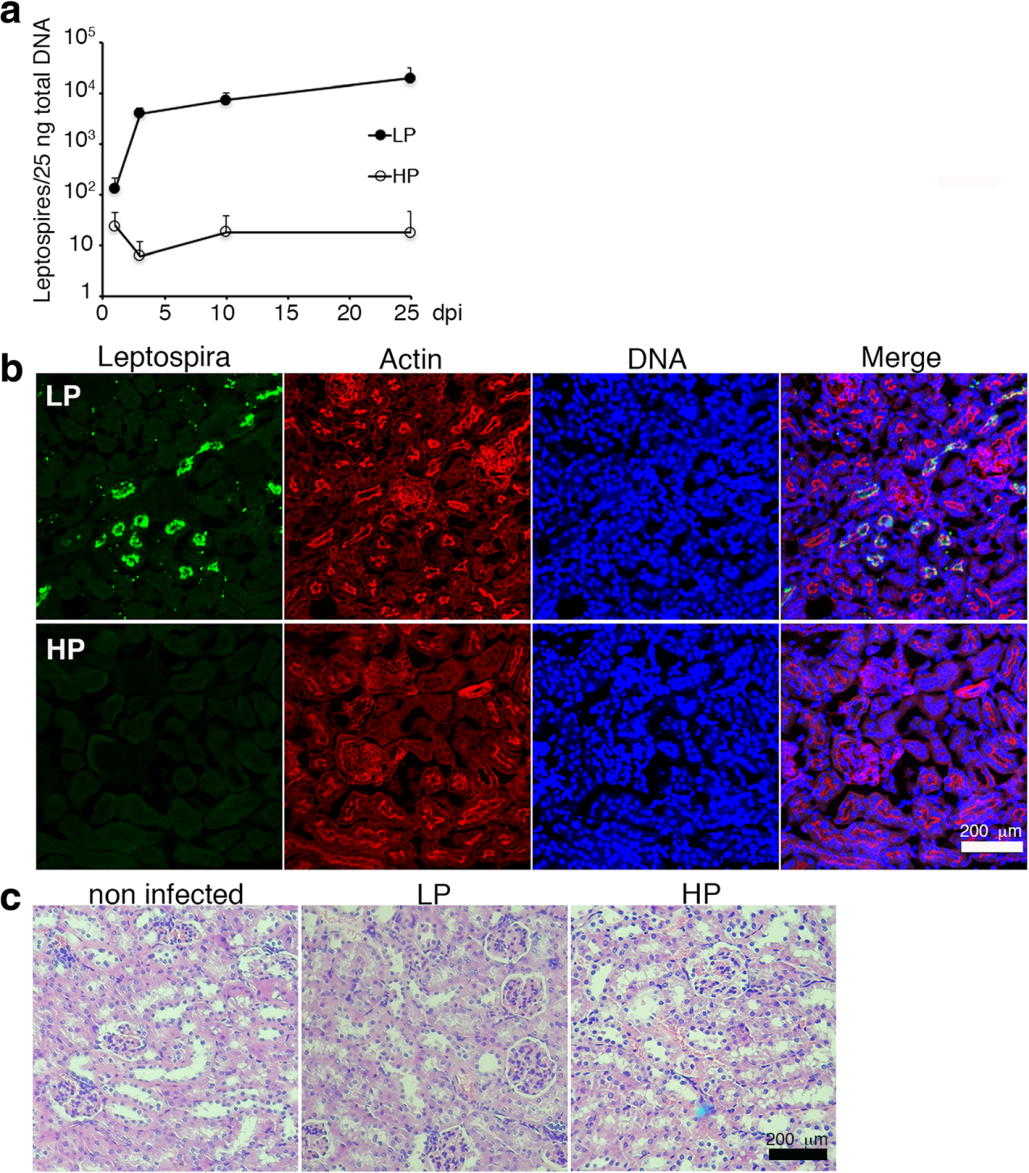
Virulent low passage (LP) strain colonizes the kidney, while high passage strain (HP) is cleared during hematogenous dissemination. **a** LP or HP strain were used to infect 5 weeks-old C57BL/6 mice. A sublethal dose of exponential growing bacteria was inoculated intraperitoneally and the presence of leptospiral DNA in the kidneys was monitored by qPCR at 1, 3, 10 and 25 days pi. **b** Immunofluorescence staining of LP- and HP-infected kidney at 25 days pi. Bacteria were detected with FITC-labeled secondary antibodies (green), actin were labeled with rhodamine-phalloidin (red) and DNA with TO-PRO-3 (blue). The merged images are also shown. **c** Histological features of kidneys from LP or HP-infected mice were compared with non-infected kidney at 25 days pi by hematoxylin and eosin staining

The kinetics of leptospiral dissemination was studied in C57BL/6 mice after intraperitoneal (IP) injection of a sublethal dose of LP or HP *L. interrogans* strains [17]. Consistent with previous reports [9, 11], the virulent LP strain disseminated and reached the kidney at 3 days post- infection (pi). The bacterial DNA load in the kidney gradually increased during the course of infection as observed by qPCR (Fig. 1a) and tubules were colonized by 25 days pi as observed by immunofluorescence (Fig. 1b). Although a hamster model of infection showed that IP injection of an HP *L. interrogans* strain resulted in the dissemination of leptospires to the kidney and colonization by 7 days pi [11], our results suggested that the HP strain was almost completely cleared during hematogenous dissemination in the mouse model of chronic infection (Fig. 1a). Histological observations of renal tissues from LP-infected mouse at 25 days pi showed normal glomerulus and tubules (Fig. 1c), which is consistent with a previous reported that compared chronic leptospirosis in hamsters and mouse models and showed no evolution of lesions in mouse kidneys [13].

### Interstitial leptospires translocated to the tubular lumen

**Fig. 2:**
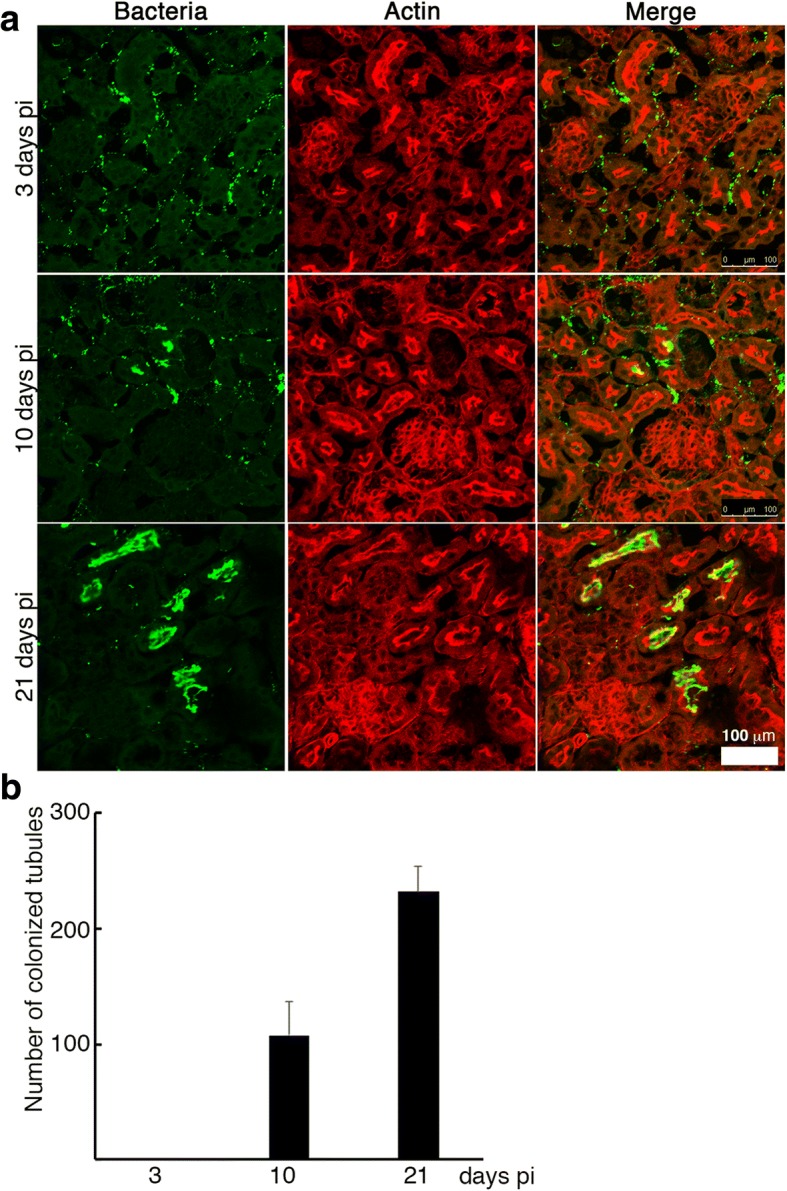
Kinetics of leptospiral dissemination in mouse infected with LP strain. Five weeks-old C57BL/6 mice were i.p. infected with a sublethal dose of exponentially growing LP strain and the kidneys were fixed at 3, 10 and 21 days pi and processed for immunofluorescence. Leptospires were stained with FITC-labeled secondary antibodies (green) and actin were labeled with rhodamine-phalloidin (red). **a** Representative confocal images. **b** The number of colonized renal tubules were quantified in 10 fields of view at × 10 magnification. Data are presented as the mean ± SD of three independent experiments

The kinetics of bioluminescent leptospires determined by live imaging analysis in mice suggest that the establishment of *Leptospira* sp. in the kidney is an early event, occurring in the very first days pi [9], although the kinetics at the renal tubule level are not well understood. We used immunofluorescence staining to identify leptospires in the kidneys of infected animals and quantified colonized tubules at 3, 10 and 21 days pi. As shown in Fig. 2a, leptospires reached the kidney at 3 days pi; bacteria were observed as single spirochetes, attached in small numbers at the basal cytoplasm of the renal tubules or aggregated in the interstitium. At 10 days pi, some leptospires were attached to the apical side of the RPTECs and by 21 days pi, there was an increase in the number of colonized tubules as well as an increase in the bacterial load in each colonized tubule (Fig. 2a and b). These results collectively suggested that during chronic infection, interstitial leptospires translocate to the tubular lumen.

### Attachment of leptospires to the epithelial brush border

**Fig. 3:**
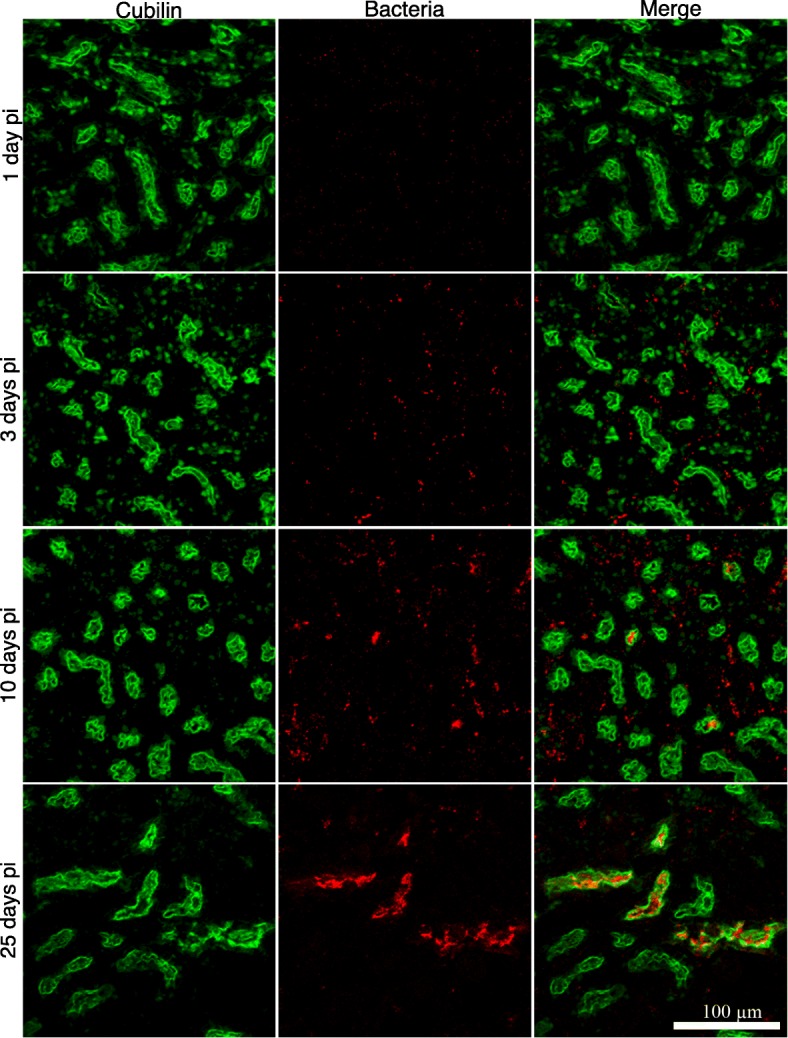
Virulent *Leptospira* colonizes a limited number of proximal renal tubules. C57BL/6 mice were infected with LP strain and the kidneys were fixed at 1, 3, 10 and 25 days pi. Cubilin (green) was stained with the TSA-Plus Fluorescein System and leptospires were stained with Alexa Fluor 647-labeled secondary antibodies (red). Representative confocal images are shown

**Fig. 4:**
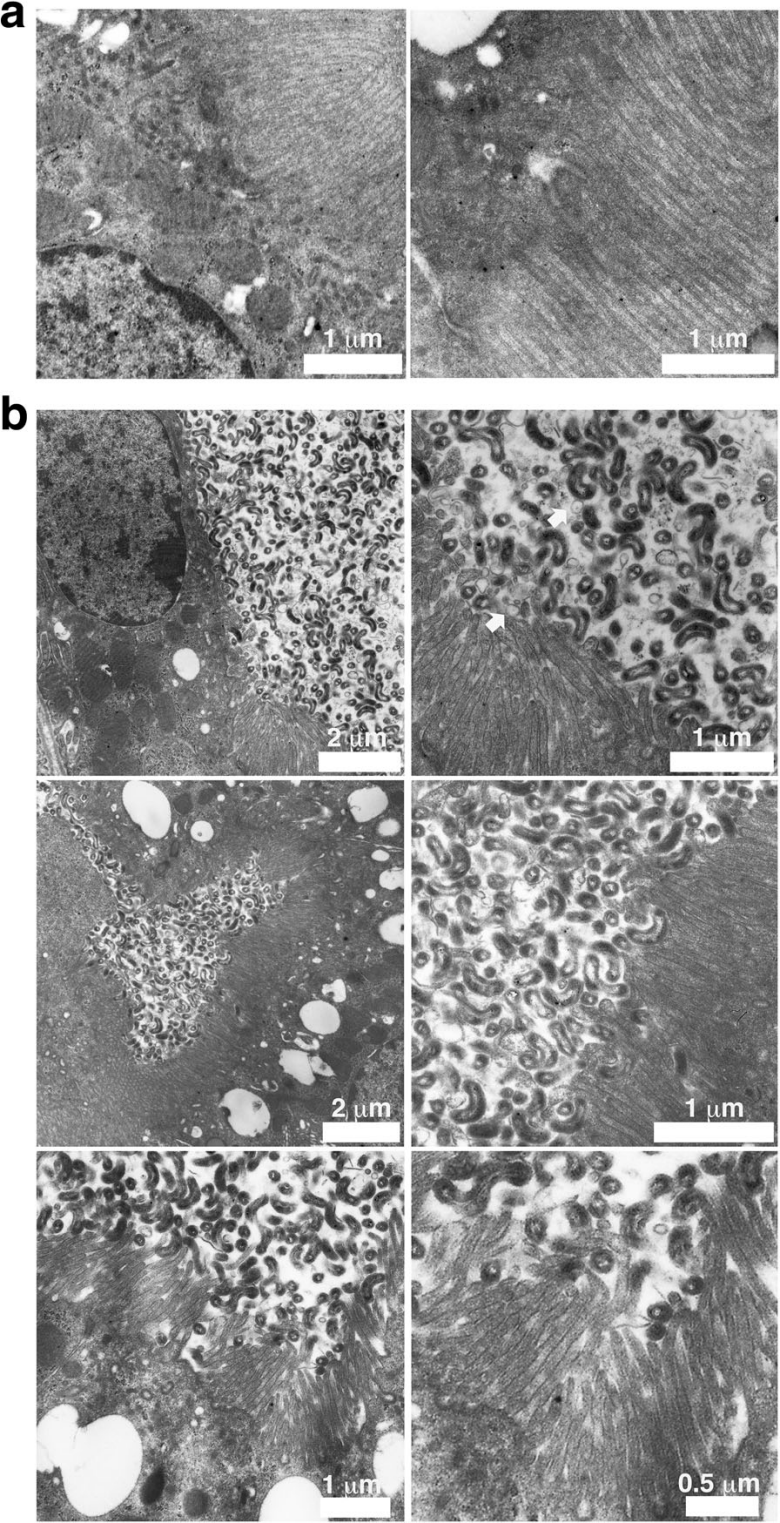
Attachment of leptospires to the epithelial brush border. C57BL/6 mice were infected with either HP (**a**) or LP strain (**b**), the kidneys were fixed at 25 days pi and processed for transmission electron microscopy. Arrows indicate membrane vesicles

To characterize the *Leptospira-*colonized tubules we dually immunostained the infected kidneys with cubilin (a receptor expressed in RPTECs) and bacteria and performed transmission electron microscopic (TEM) analysis of the infected kidneys. As shown in Fig. 3, leptospires gradually colocalized with cubilin during the course of the infection. TEM analysis of kidney sections showed that the tubular lumen was completely filled with aggregated leptospires interacting with each other directly or through membrane vesicles. Heavily colonized epithelial cells did not show any sign of cell death, but the organization of the brush border in LP-infected kidneys was altered when compared with HP-infected kidney (Fig. 4a and b). Collectively, these results suggested that during tubular colonization, aggregated leptospires formed a biofilm-like structure to maintain a protective and replicative niche in a limited number of RPTs.

### Delayed oxidative stress response in LP-infected cells

**Fig. 5:**
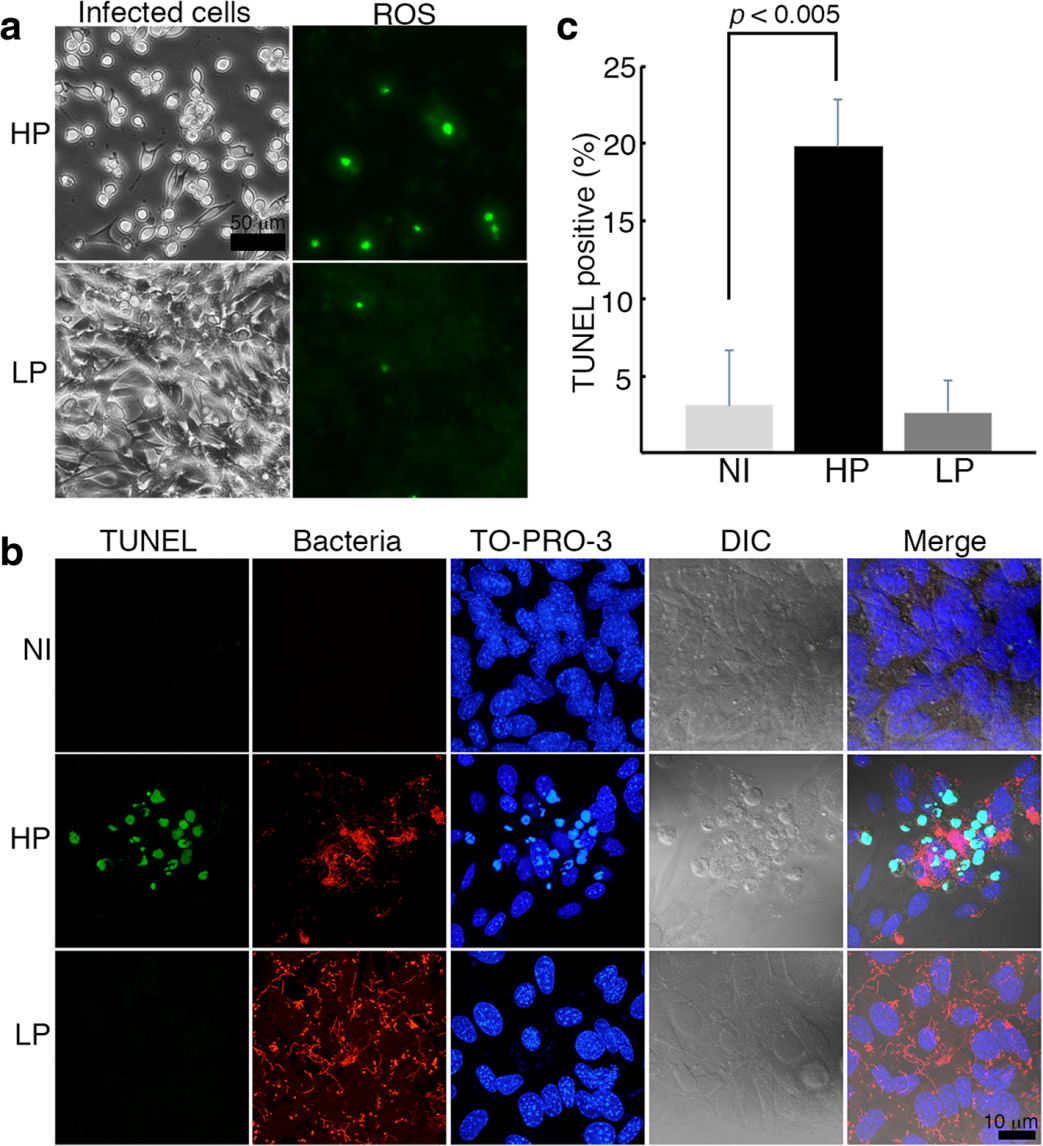
Delayed of ROS production in LP-infected cells. TCMK-1 cells were infected either with LP or HP strain. **a** Detection of intracellular ROS. The nuclear green fluorescence reflects ROS. (**b** and **c**) Non-infected (NI) and infected cells were fixed and processed for *Leptospira* immunostaining and TUNEL staining (green) at 48 h pi. **b** Representative confocal images. Total bacteria were detected with TRITC-labeled secondary antibodies. DNA was stained with TO-PRO-3 (blue). The merged image of immunofluorescence and differential interference contrast (DIC) are also shown. **c** Quantification of TUNEL positive cells. Data are the mean ± SD of three independent experiments

The adherence of exogenous components, such as crystals, to RPTECs during kidney stone disease, can cause various cellular reponses including the production of reactive oxygen species (ROS) and the stimulation of signaling molecules [19]. To better understand the effect of *Leptospira* infection on RPTECs, we used an in vitro model of RPTEC infection. Infection of the mouse RPTEC line, TCMK1, showed that adherence of leptospires to RPTECs induced an increase in intracellular ROS (Fig. 5a) and cleavage of DNA as shown by TUNEL staining (Fig. 5b and c). This oxidative stress response was delayed in LP-infected cells.

**Fig. 6:**
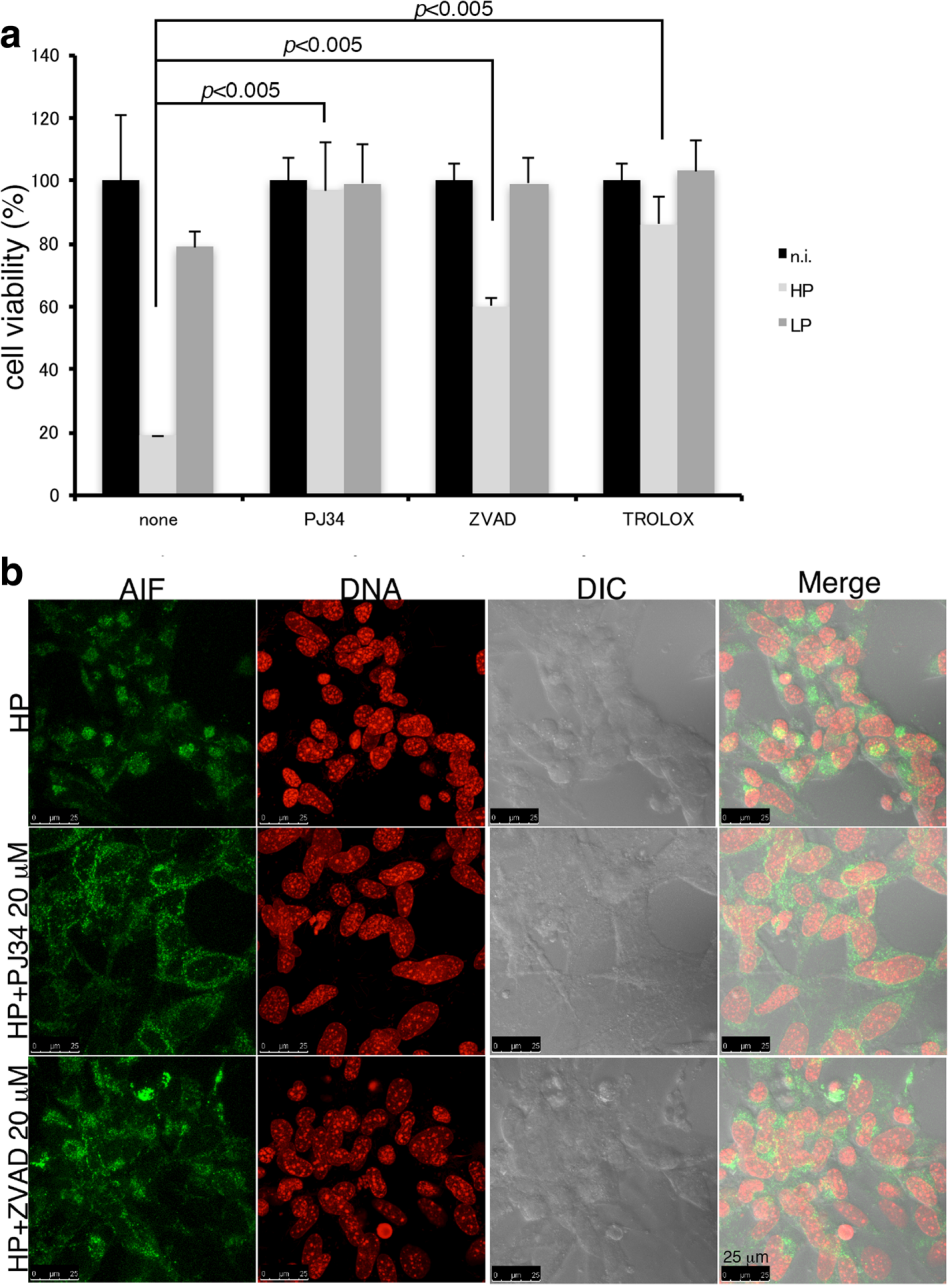
Inhibition of the oxidative stress response confers protection to HP-infected TCMK-1 cells. TCMK-1 cells were pre-treated with different inhibitors and infected with either HP or LP strain for 48 h. **a** Cell viability was determined by crystal violet assay and shown as the mean percentages ± SD of three independent experiments. **b** Cells were fixed and stained for AIF. Representative confocal images shown nuclear translocation of mitochondrial AIF in HP-infected cells, which is inhibited by the PARP-1 inhibitor, PJ34

Oxidative stress can cause DNA breaks which induce the activation of the DNA nick sensor enzyme poly(ADP-ribose) polymerase-1 (PARP-1). PARP-1 is an activator of caspase-independent cell death. Overactivation of PARP-1 initiates a nuclear signal that propagates to mitochondria and induces the release of the apoptosis-inducing factor (AIF) [20]. Thus, we next investigated if PJ34 [a Poly (ADP-ribose) polymerase-1, PARP-1 inhibitor] or ZVAD-FMK (a pan-caspase inhibitor) can inhibit cell death induced by HP. As shown in Fig. 6a, cell death was inhibited by PJ34 and partially inhibited by ZVAD-FMK. Cell death was also inhibited by the anti-oxidative compound TROLOX (Fig. 6a). Moreover, the nuclear translocation of mitochondrial AIF was observed in HP-infected cells that was inhibited by PJ34 (Fig. 6b). These results suggested that leptospiral infection of RPTECs induced a host stress response which involves PARP-1 activation, followed by AIF nuclear translocation and cell death. However, virulent leptospires possess some mechanism/s to counteract this host response to create a safe niche for their long-term colonization.

## Discussion

This is the first study to analyze the kinetics of leptospiral colonization of the proximal renal tubule in a mouse model of chronic infection. C57BL/6 animal model was used in this study as a chronic model of infection because it was shown by Ratet et al. to exhibit biphasic disease with a self-resolving hematogenous dissemination followed by renal colonization [9]. Thus, this model is suitable to study renal colonization mechanisms. Moreover, the availability of knocked out mice on a C57BL6/J background will allow the analysis of host factors involved in kidney colonization in the future. Our results suggested that leptospires become attached at the basal cytoplasm of RPTECs before migrating from the interstitial space to the lumen side. These results are in agreement with previous observations by Marshall [21] and Barocchi et al., that suggested that leptospires rapidly translocated in polarized epithelial cell monolayers [22]. By 25 days pi leptospires reside mainly in cubilin-positive RPTs. Interestingly, the number of colonized tubules is limited and some tubules are devoid of leptospires. Our results suggested that the activation of signals that allow the bacterial replication and tubular colonization requires a threshold of leptospiral infective dose in each RPT.

Biofilms are structured communities of adherent microorganisms composed of a complex extrapolymeric substance matrix, DNA and membrane vesicles [23]. Biofilms represent a microbial survival strategy, where microorganisms exist in a dynamic equilibrium to form clusters of bacteria [24]. Saprophytic and pathogenic leptospires are able to form biofilms in vitro and it was suggested that this ability might help the bacteria to survive in environmental habitats and to colonize the hosts [25]. In vivo leptospiral aggregates were observed in placental vessels from infected pregnant guinea pigs [26]. On the other hand, membrane vesicles production by *Leptospira* have been reported in vitro in response to environmental stressor [27–29]. Our TEM analysis showed the presence of MVs in a biofilm-like structure within the tubules. Environmental changes in the renal tubules such as the release of host factors and low iron, induce the transition to a biofilm lifestyle that can cause the modulation of MV production [30]. Therefore, our observation suggests that to survive within tubules and/or interact with RPTECs leptospires are induced to produce a biofilm-like structure. Recently, Turnbull et al. reported that explosive cell lysis is a mechanism for the biogenesis of bacterial membrane vesicles and biofilms in *Pseudomonas aeruginosa* [23]. Thus, the MVs that we observed might also be remnants of dead leptospires. Although detachment of bacteria from biofilms has been considered as a passive behavior for long time, some reports suggested that it can be an active process that allow bacteria to colonize new niches before nutrients become limited [24]. Intermittent excretion of leptospires into the urine in chronically infected animals might be the result of biofilm detachment from the tubules and the excretion of bacteria as a complex aggregate might confer advantages to survive in the environment. Further studies will elucidate the possible association between biofilm formation in renal tubules and the pathogenic cycle of *Leptospira.*

The comparison of LP and HP *Leptospira* strains has been used to identify virulence-associated genes in several studies [17, 31–33]. Ballard et al. reported that virulent leptospires attached to RPTECs, while the avirulent variant of a serovar Copenhageni strain did not adhere to epithelial cells at all [34]. In this study, we found that the avirulent variant of a serovar Manilae strain adhere to RPTECs. Therefore, our data suggested that the capacity to adhere to epithelial cells is not correlated with virulence.

Although HP was cleared during hematogenous dissemination in the chronic model of infection used in this study, the infection of TCMK1 cells suggested that the manipulation of host oxidative stress response is a mechanism used by the virulent strain to maintain a protective and replicative niche in RPTs during chronic infection of kidneys. Microbial infection can trigger several host cell responses such as oxidative stress, mitochondrial stress and DNA stress which activate antimicrobial defense systems. The activation of these stress responses can also lead to cell death [35]. However, pathogenic bacteria are able to manipulate the host cell reponses in order to promote the long-term colonization of epithelial cells [36, 37]. Oxidative stress that occurs during the host defense response following infection with *Leptospira* spp. have been reported to be regulated by the peroxide sensor and the transcriptional regulator PerR [38, 39]. Moreover, catalase is required for the virulence of *Leptospira* spp. in an acute model of infection [6], suggesting that the inhibition of oxidative stress response might be a strategy used by *Leptospira* in the acute as well as the late stage of infection. We found that cell induction by HP leading to nuclear translocation of AIF is PARP-1 dependent. The release of mitochondrial AIF has been reported as a mechanism of *Leptospira*-induced apoptosis in macrophages through a caspase-8- dependent pathway [40]. Thus, different signal pathways are activated in response to *Leptospira*-infection during the course of infection and the understanding of the interactions between *Leptospira* and the different cell types will help to achieve a better control of leptospirosis.

## Conclusion

Our results suggested that after escaping the blood defenses, leptospires create protective and replicative niches in the base membrane and luminal sides of RPTECs by manipulating the host oxidative stress response and forming a biofilm-like structure. During the long-term colonization, leptospires aggregated and attached to the brush border and membrane vesicles which are involved in the formation of a biofilm-like structure.

## Abbreviations

AIF: Apoptosis-inducing factor
HP: High passage
LP: Low passage
PARP-1: Poly (ADP-ribose) polymerase-1
pi: Post infection
qPCR: Quantitative real-time PCR
RPTEC: Renal proximal tubule epithelial cell
TEM: Transmission electron microscopy
